# Does the Decline in Caries Prevalence of Latin American and Caribbean Children Continue in the New Century? Evidence from Systematic Review with Meta-Analysis

**DOI:** 10.1371/journal.pone.0164903

**Published:** 2016-10-21

**Authors:** Thais Gimenez, Beatriz Albuquerque Bispo, Daniela Pereira Souza, Maria Eduarda Viganó, Marcia Turolla Wanderley, Fausto Medeiros Mendes, Marcelo Bönecker, Mariana Minatel Braga

**Affiliations:** Department of Pediatric Dentistry, School of Dentistry, University of São Paulo-USP, São Paulo, Brazil; University of Florida, UNITED STATES

## Abstract

**Objective:**

To carry out a systematic review with meta-analysis of prevalence of caries in Latin America and Caribbean children considering studies performed in this new century.

**Methods:**

Two reviewers searched PubMed, Embase, LILACS and governmental databases through May 2016 to identify papers published in English, Portuguese or Spanish. Studies in those countries performed with 5–6 or 11–13 year-old children and that presented separate prevalence figures from primary and permanent teeth were selected. We performed a descriptive analysis of studies and meta-analysis to calculate the overall prevalence and 95% confidence intervals (95% CI) in both primary and permanent teeth. We also analyzed the trends of prevalence of caries through the years and influence of other variables on caries prevalence using multilevel analysis.

**Results:**

Seventy-five studies were included from the 1,306 articles initially retrieved. The meta-analysis of caries prevalence grouped for Latin American and the Caribbean countries were highly different from Brazil and other investigated countries for primary teeth (5–6 years-old—Brazil: 0.52, other countries:0.70) and permanent teeth (11–12 years-old—Brazil: 0.56, other countries: 0.63). For studies conducted only in Brazil the prevalence was significant lower for primary but not for permanent teeth. In Brazil, a downward trend of caries prevalence was observed in 11-13-year-old children.

**Conclusion:**

Despite the decline of caries prevalence in permanent teeth, mainly in Brazil, the disease still affects more than half of the children population in Latin American and Caribbean countries in the 21^st^ Century.

## Introduction

The decline of caries experience in several countries has been accompanied by a process of inequality in the distribution of the disease in children and adolescents [1–4]. Studies have shown trends of high prevalence of caries in a minority of the population, which remains on the fringes of society. Epidemiological surveys of dental caries can provide support for the creation of public health policies. They allow detecting the proportion of the population which is affected by the dental caries, as well to recognize groups of higher risk to develop the disease.

In 2003, Bönecker and Cleaton-Jones performed a systematic review of articles published between 1970 and 2000 about trends in dental caries in Latin America and the Caribbean in 5–6 and 11–13 years old children [5]. The Latin America and Caribbean is a region defined by the United Nations Children's Fund (UNICEF) and comprises developing and underdeveloped countries with similar geographic, cultural and socioeconomic characteristics [6]. The authors found a decrease in the prevalence of the disease in newer studies performed in this area, that it was less prominent in the late 90s [5].

On the other hand, the apparent decline in caries prevalence does not mean that this is no longer a public health problem [5]. Furthermore, it is not clear if the reduction in caries prevalence is still occurring in this new century. In order to facilitate when addressing actual public health policies, we aimed to carry out this systematic review of studies aiming to demonstrate the trends in caries prevalence in Latin America and the Caribbean in 5–6 and 11–13 years old children, considering only studies performed in this new century. We also performed meta-analyses to calculate overall prevalence and to investigate the tendency of dental caries along the years in this specific region of America.

## Materials and Methods

This paper was prepared according to “Meta-analysis of observational studies in epidemiology” (MOOSE) guidelines [7]. The MOOSE checklist is presented as Supporting Information (S1 Table).

### Information sources

We performed the literature search in MEDLINE (PubMed), Embase and LILACS for articles published until May 23^th^, 2016 that reported prevalence of dental caries in 5–6 and 11–13 years old children. LILACS is the most important and comprehensive database covering the scientific literature of Latin America and Caribbean region. Moreover, we checked governmental websites of all Latin American and Caribbean countries that could provide data about official epidemiological surveys about dental caries. The references of the articles included were also checked for verification of possible items not identified by the search. No restrictions were made with respect to the study design.

### Search Strategy

**Fig 1:**
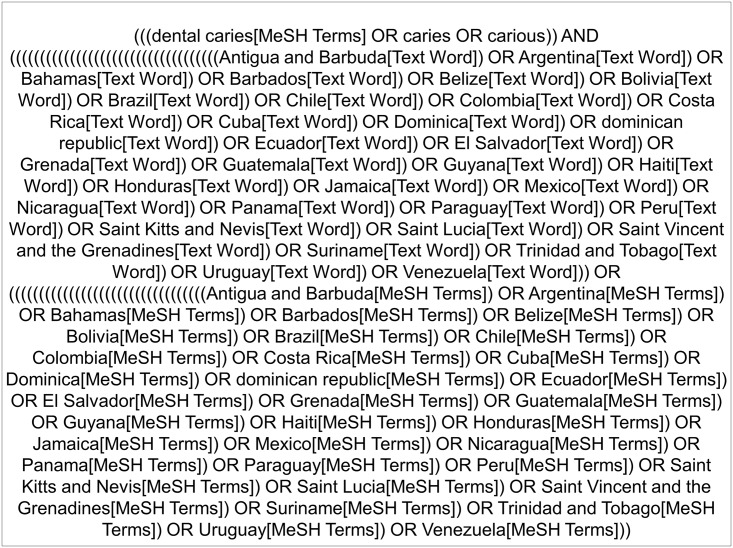
Search strategy. Chart containing the search strategy for electronic databases.

The syntax was developed by the research group to search in the MEDLINE database and was adapted for other databases. Search strategy is presented in Fig 1.

The results of searches of various databases were cross checked, in order to locate and eliminate duplicates.

### Study Selection and Eligibility criteria

After locating the studies, the titles and abstracts were examined to ensure they fulfilled the following inclusion criteria. We included abstracts which mentioned that (1) population had been clearly defined as 5–6 and/or 11–13 years old children (2) assessment of dental caries; (3) study was conducted in Latin America and the Caribbean countries and (4) the manuscript was published in the English, Portuguese or Spanish languages.

The articles whose titles and abstracts met these inclusion criteria were then fully read. Then, we maintained for data collection and analyses those manuscripts that: (1) presented the data about prevalence of dental caries, (2) presented separate data from each age group, as well as from primary and permanent dentition; (3) reported primary results of in vivo original research; (4) collected separately data about cavitated or non-cavitated lesions; (5) used a minimum sample of 30 individuals from the studied population [5] and (5) collected data after 2000’s. If the study did not report the year of survey, we considered the year of publication, supposing if there is no mention about the period of data collection, we were including a recent study by the occasion of the publication.

Studies in specific groups (indigenous or socio-excluded/marginalized populations, patients with special needs, immunocompromised, hospitalized or institutionalized patients etc.) were also excluded. If different studies had been conducted in the same sample, the less complete study was also excluded.

Attempts to access the studies were done through internet, search in the library of the School of Dentistry, University of São Paulo and other libraries participating in the information sharing system. Papers which could not been assessed by these strategies were also excluded from our sample.

Three investigators (TG and MMB) independently identified potential references and eliminated irrelevant studies. Another investigator (MEV) collaborated with them in updating the results after the initial search. Doubts or disagreements were solved by discussion with a third researcher (FMM).

### Data collection process

Data were extracted by two reviewers (TG and MMB) directly from the full texts of articles to structured tables containing all variables. A second researcher (FMM) independently verified the extracted data. Discrepancies were solved by checking the source and conjoint discussion. Both reviewers were able to read and understand the papers in all included languages. The interexaminer agreement ranged from 0.93 to 0.98.

From selected papers, caries prevalence rates were collected, considering only the cavitated caries lesions (considering as cavitation any discontinuity of enamel or dentine exposure [8]. Even if studies presented prevalence of non-cavitated caries, these data were not considered in our analyses. In cases in which studies reported data from primary teeth in other age group than 5–6 years-old, these data were not considered for our analysis. The same is valid for permanent teeth in different ages than 11–13 years-old.

The following information was also extracted from papers: title, authors, year of publication and data collection, country, prevalence of dental caries, children’s age, number of children involved, criteria used for caries diagnosis and if examiners were calibrated or not.

### Summary Measures and synthesis of results

The statistical analyses were performed separately at two different categories: primary and permanent teeth. The analyses were performed for all countries. Due to the plentiful contribution of Brazilian studies in this field, we also performed sub-group analyses, for Brazil and other countries, separately.

We tested the heterogeneity among the studies using the Cochran Q and I-squared tests. The Cochran Q test was used to verify whether heterogeneity is present, while the I-squared test was used for assessing the percentage of the variability in estimates. Finally, bias was graphically represented by funnel plots and their asymmetry was tested by Egger’s test. All these statistical approaches were used by both sub-groups using the StatsDirect software (StatsDirect Ltd, Cheshire, UK) and R software version 3.3.1 (R Foundation for Statistical Computing, Vienna, Austria).

For summarizing the results, firstly, statistical pooling of caries prevalence and its respective 95% confidence intervals (95%CI) were carried out. Using random effect models, we also test the subgroup differences between the pooled results considering the country in which data collection had been done—Brazil or other countries (R software version 3.3.1 (R Foundation for Statistical Computing, Vienna, Austria).

Afterwards, we verified the trend of caries prevalence along the years. We also evaluated the influence of other factors on caries prevalence: country (Brazil vs. other countries); indices for caries detection used (WHO vs. other indices); sample size (studies performed up to 100 subjects (small); studies performed with more than 100 and up to 500 subjects (medium); studies performed with more than 500 subjects (large)). For these analyses, we used multilevel Poisson regression analysis and we calculated the prevalence ratio (PR) and 95%CIs. Multilevel analysis was used considering two levels: children examined in each study (1^st^ level) and different studies (2^nd^ level). We carried out the multilevel analysis deriving individual data of each participant based on reported caries prevalence, considering as the outcome variable children with no caries or with caries lesions. However, all explanatory variables were related to the second level, since it was not possible to gather explanatory variables related to the children from all primary studies. For the multilevel analyses, Stata software, version 13.1 (StataCorp LP, College Station, Texas, USA) was used.

## Results

### Study Selection

**Fig 2:**
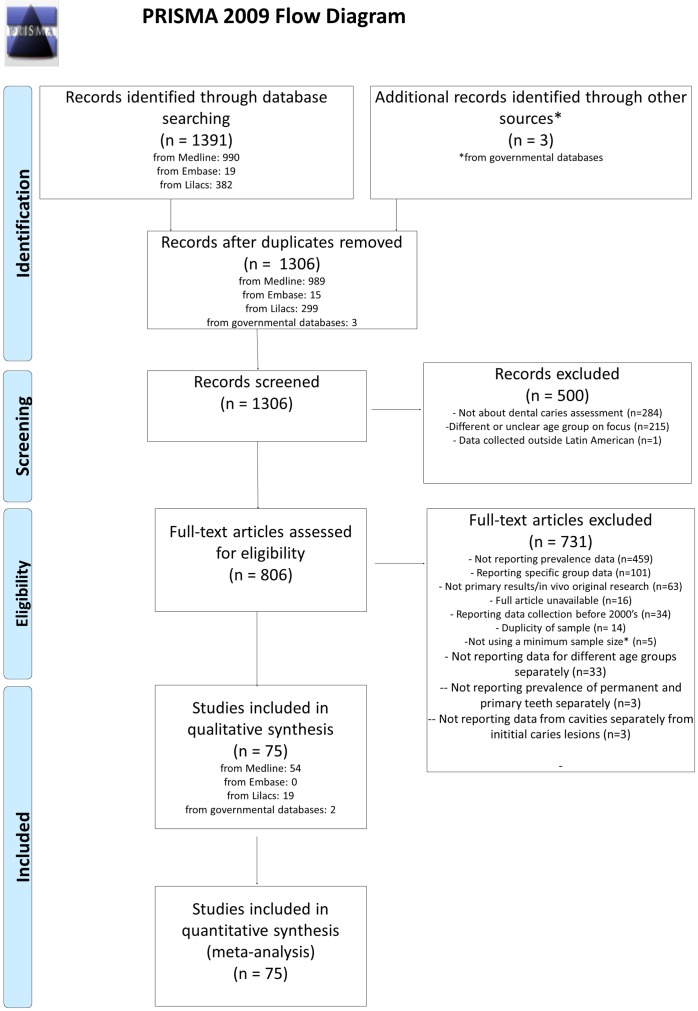
Flow diagram for selection of studies (*according to the previous systematic review published by Bonecker et al., 2003 [3]).

Study selection flow is shown in Fig 2. Medline (PubMed), Embase LILACS and governmental databases searches yielded 1,391 studies (Fig 2). Using Medline as reference, 85 articles were excluded due to duplication. Thus, the three databases identified 1,306 unique studies. On the basis of title and abstract, we excluded a further 500 articles. Seven hundred thirty-one articles were excluded after reading full text, due to reasons detailed in Fig 2. Finally, 75 studies remained for the analysis.

### Study Characteristics

Publication year ranged from 2000 to 2016. The most recent data collection had been in 2014. The vast majority of studies were conducted by calibrated examiners using WHO criteria for caries diagnosis in permanent teeth in Brazil. Brazilian studies corresponded to 70–80% of the studies included in this review. Two studies, one with primary and other with permanent teeth were conducted using the International Caries Detection and Assessment System (ICDAS). Besides Brazil, Mexico was the following country that most studied caries prevalence in children (3 studies in primary teeth and 5 studies in permanent teeth). Similar trend was observed among those titles excluded from the analysis by not accessibility (Fig 2).

Characteristics of each included study including primary and permanent teeth are summarized in S2 and S3 Tables, respectively.

### Bias and heterogeneity assessments

**Fig 3:**
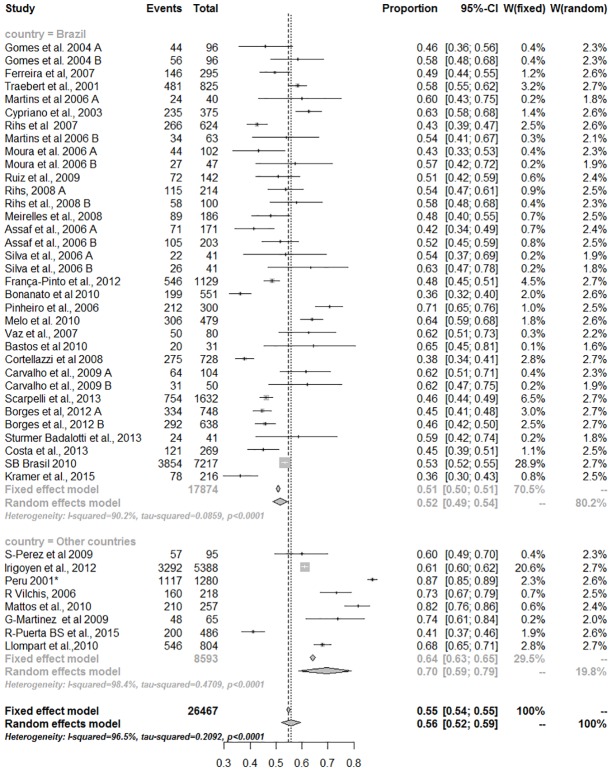
Meta-analysis for caries prevalence in primary teeth (Heterogeneity—Q Cochran: p<0.0001; I-squared = 94.5% (95% CI = 93.6% to 95.3%).

**Fig 4:**
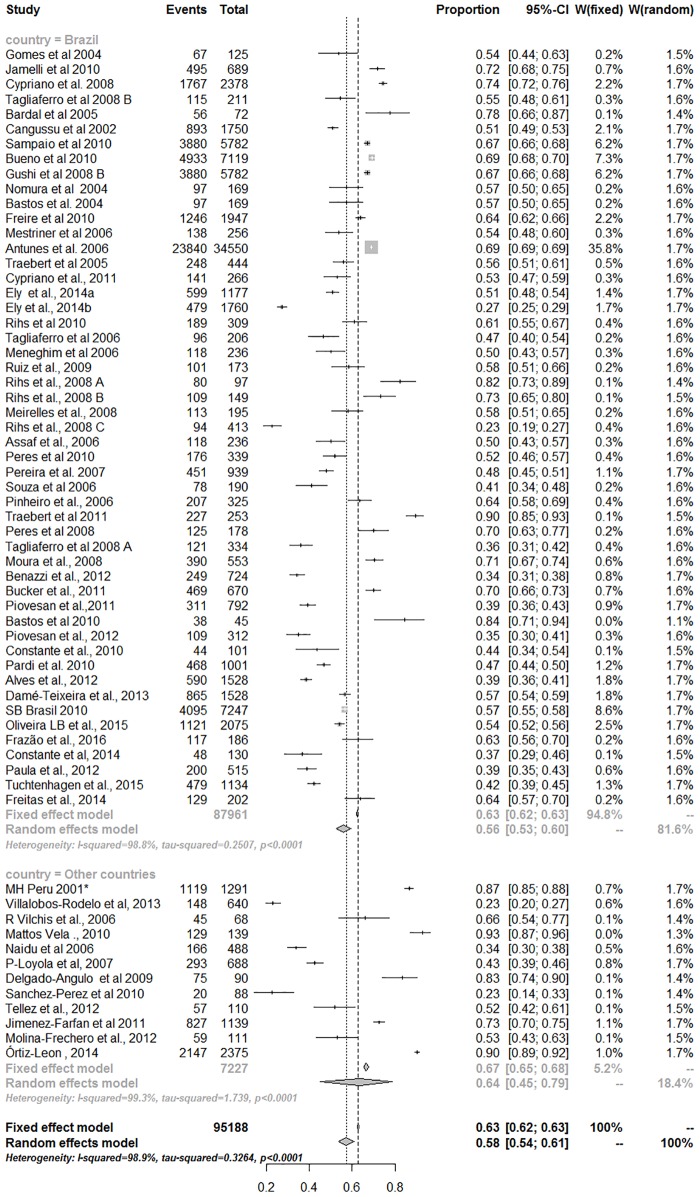
Meta-analysis for caries prevalence in permanent teeth (Heterogeneity—Q Cochran: p<0.0001; I-squared = 98.8% (95% CI = 98.7% to 98.8%).

A high percentage of heterogeneity was observed among the studies, both for analyses performed with primary and permanent teeth, despite Brazil or other countries were considered (Figs 3 and 4).

**Fig 5:**
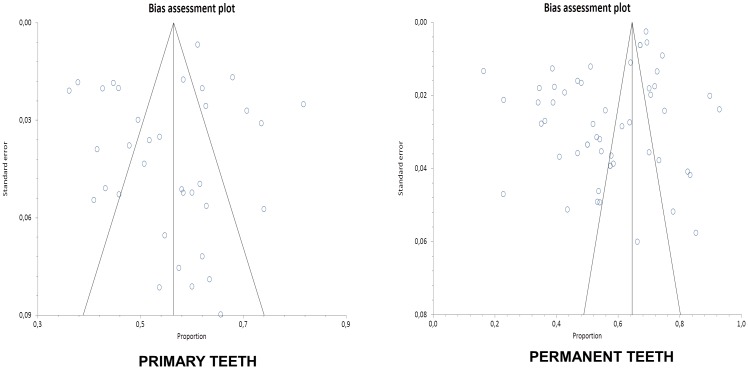
Bias assessment plots. (a) studies with primary teeth and (b) studies with permanent teeth.

The Egger’s test revealed any source of bias for studies performed in permanent teeth (bias = -5.55; 95% CI = -8.90 to -2.19—p = 0.002), but not for primary ones (bias = -0.94; 95% CI = -3.44 to 1.55—p = 0.45). The bias assessment plots illustrate the asymmetry among prevalence studies of dental caries in permanent teeth (Fig 5).

### Synthesis of results

Data related to the primary dentition was obtained from 47 papers totaling 26,467 children who were examined in these studies. A significant difference in pooled prevalence figures were observed between Brazilian surveys and those performed in other Latin American and Caribbean countries (p = 0.002, Fig 3).

**Fig 6:**
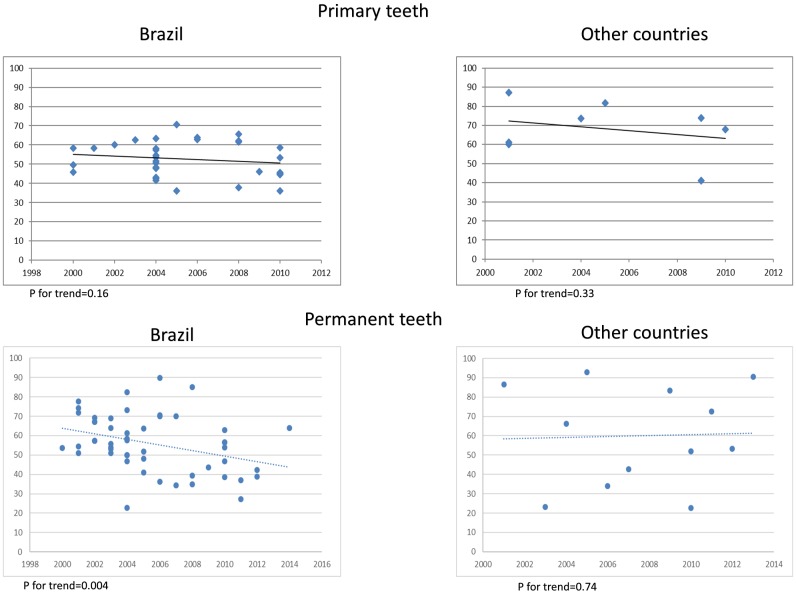
Trend charts for caries prevalence.

The meta-analysis including only Brazilian surveys in primary teeth showed approximately 20% lower pooled prevalence than other studied countries a 10% higher prevalence pooled rates were found (Fig 3). Complementarily, in the multilevel analysis, prevalence of dental caries in the primary dentition was significant higher in other Latin American and Caribbean countries compared to the Brazilian figures (Table 1). A trend of not changing along the years was observed for primary teeth independently of subgroup region studied (Brazil, Fig 6A or other countries, Fig 6B).

**Table 1:** Influence of some characteristics related to the different studies on caries prevalence.

Characteristics	Primary teeth		Permanent teeth	
	PR	95% CI	PR	95% CI
**Year**				
Number of years after 2000 (continuous variable)	0.98	0.96 to 1.00	0.98	0.96 to 1.00
**Country (ref.: Brazil)**				
Other countries	1.32 *	1.14 to 1.52	1.02	0.83 to 1.25
**Indices (ref.: WHO)**				
ICDAS	1.37	0.84 to 2.23	1.41	0.91 to 2.20
**Sample size (ref.: up to 100 subjects)**				
Among 101 and 500 subjects	0.92	0.77 to 1.09	0.78	0.61 to 1.03
More than 500 subjects	0.87	0.73 to 1.04	0.83	0.64 to 1.07
**Examiners’ calibration (ref. without calibration)**				
With calibration	0.87	0.53 to 1.41	0.97	0.93 to 1.27

* statistically significant association (p < 0.05)

PR = prevalence ratio; 95% CI = 95% confidence intervals.

For the permanent dentition, analyses were based on 86,358 patients who participated in 63 different studies. The meta-analysis showed an overall caries prevalence close to 60% (Fig 4). Although, there is no statistical difference on pooled prevalence between region subgroups for permanent teeth (p = 0.42), significant decreasing trend in caries prevalence was observed only in Brazil (Fig 6C and 6D). The caries prevalence in permanent teeth in Brazil decreased, on average, 3% per year after 2000 (PR = 0.97; 95% CI:0.95 to 0.99).

Both of studies that used ICDAS for caries detection were conducted in Colombia and showed prevalence values, considering cavitated lesions, similar to other studies. The sample size of the study did not influence the prevalence values either (Table 1).

## Discussion

The decline of caries prevalence in the new century in Latin American and the Caribbean is still occurring among Brazilian children. However, the decrease in these prevalence rates seems to be mainly related to permanent dentition in children aged 11 to 13 years. A scarce contribution of other counties could be evidenced in this review to explain the prevalence rates in the studied region. Besides, we noticed different trends in Brazil compared to other countries included in the same geo-cultural region.

A previous systematic review also examined the prevalence of caries in children in these age groups both in Latin America and the Caribbean as well as in Sub-Saharan Africa, the Middle East and North Africa [9]. Rates decreased over time in Latin America and the Caribbean and have remained almost static in other regions [9], what reinforces the apparent decline in caries prevalence does not mean that this is no longer a public health problem [5]. Similarly, we continued to observed this decline among Brazilian 11-to-13-year-old children, but not among other groups. Therefore, understanding what may be occurring differentially in these populations is really needed to guide further policies.

Systematic reviews of prevalence studies may be useful to support the construction of public health policies. Although pooled prevalence rates could summarize the overall condition of prevalence of dental caries in a specific population, as Latin American and Caribbean, the graphic representation of all studies in the Forest plot also permit to observe the participation of each primary study in the pooled results, evidencing possible outliers, which could be carefully investigated in their particularities.

Due to individual characteristics of different studies and studied populations, a high level of heterogeneity may be expected in a meta-analysis of observational studies [10]. If the heterogeneity may be caused randomly, it can be modeled [11]. That is why we used the random models, which produced wider confidence intervals [11] and whose inferences may be generalized to the pool of studies from which the sample (included papers) was obtained, considering the differences among studies as a process of random sampling [12]. We also used the subgroup analyses for exploring the heterogeneity. A priori, we have decided to analyze primary and permanent teeth separately due to referring to different and particular age groups. A posteriori, we also performed subgroup analyses for Brazil and other countries, since expressive difference in number of publications was found. Despite that, as prevalence studies involve several particularities not from the design but from the population by itself, the high level of heterogeneity could not be reduced.

In the preset study, subgroup analyses mostly contributed to permit identifying the necessity of directing different preventive strategies to different age groups, since they presented different patterns. It is evident a higher number of children are being affected by dental caries during early childhood compared to adolescence. Additionally, differences between areas inside this socio-geographic region could also be addressed. On the other hand, we should have caution in considering findings from subgroup analyses. Although subgroup analyses are important to comprehension of some particularities, as discussed above, they have less statistical power than the global analysis and extrapolations should be made carefully [13].

Despite prevalence rates in adolescence are decreasing in Brazil, they still considered high when compared to the Scandinavian countries, which have achieved low levels of caries experience across the time [14, 15]. Indeed, we can point out that more than half of 12-year-old adolescents have caries in the Latin American and Caribbean. Indeed, this region is also responsible for still higher levels of caries experience than other continents, as North America and Europe [16]. These high rates of caries prevalence are even more worrisome, especially considering the age of these children, the short time that these teeth are present in the oral cavity and how longer they will be still in risk of caries progression.

With regard to the primary teeth, we did not found a significant decrease in caries prevalence along the years in studies conducted after 2000. In fact, in childhood, the age of 6 was found as a peak for global untreated caries [16]. In Brazil, two more recent national surveys on dental caries, performed in 2003 and 2010 showed similarity in caries prevalence of 5-year-old children [17, 18]. A possible explanation for this situation could be the low rates of oral health assistance probably offered to preschool children [19]. A recent epidemiological survey showed that less than 25% of preschool children in a Brazilian city had already visited a dental office [20]. We also speculated that the resistance of some clinicians and researchers in recommending the evidence-based widespread use of fluoride dentifrices with at least 1,000 ppm F for preschool children [21] could contribute for this scenario.

The results found for primary teeth in other countries seem to be even more disappointing. The caries prevalence in primary dentition in these countries was around 20% higher than in Brazil. The studies performed in other Latin American and Caribbean countries included in this systematic review demonstrate the small amount of epidemiological surveys in children, especially with regard to the primary dentition. Maybe, the small number of studies in these countries may be explained by the difficulty in conducting the examinations in young children compared to older children and adults. It is noticeable; however, Brazilian concern in this sense, since the vast majority of studies of primary teeth was performed in this country. This scarceness of non-Brazilian studies hampers the extrapolation of data obtained, since the few included articles are from limited countries.

Most of the South American continent and the Caribbean have no published data on this topic or about the age groups studied here. Thus, it is difficult to analyze how caries is behaving in these other locations and generalize trends could not be correct. Although a limitation of our study has been the exclusion of not accessible papers by the time this review was performed, based on a preliminary analysis of excluded titles, we expected the same representativeness of Latin American countries as observed for the collected data.

Other interesting finding is concerned the indices used for caries detection. Studies which used ICDAS instead of WHO criteria presented similar figures when cavities were considered. This correspondence was previously observed in primary studies [8, 22, 23], other authors do not agree with this similarity [24]. However, since few studies using ICDAS were included in our systematic review, additional research should be conducted to clarify this issue.

Although some asymmetry has been detected for studies including permanent teeth, we believe it is more prone to be an effect of the different population studied than properly a publication or selection bias. Even considering publication bias could be often related to heterogeneity among studies [11], due to dealing with identified bias, we adopted the meta-regression analyses considering the sample size used in surveys [25]. Different sizes of studies did not present significant differences in the present review, indicating that the trend of caries prevalence is similar since a minimum sample has been collected. Actually, the observed asymmetry in plotting the prevalence rates from studies with permanent teeth was intimately related to some Brazilian studies performed in more developed areas of the country. Indeed, small surveys could be different from larger ones in nature, because of intrinsic characteristics [25]. These studies may indeed reflect overall improvement in living condition and also, higher concentration of research centers in Latin America and Caribbean region.

The statistical analysis used to evaluate the differences in caries prevalence along the years and influence of other variables, such as country, index used to detect caries lesions and size of the study was innovative. Systematic reviews represent a clear example of clustered data (subjects of the research are clustered in the different studies). Therefore, multilevel analysis is a statistical approach to deal with this type of data [26]. Using this approach, we could create a database with the number of participants of each study who had dental caries or not. This procedure was possible, however, due to the simplicity of the outcome, which could be easily derived from prevalence and sample size of each sample. However, it was not possible to analyze associations with explanatory variables related to the participants of the studies, such as sex, socioeconomic data or other examples.

Analyzing the data from Brazilian children, the analysis of caries prevalence in permanent teeth suggests that something is actually being done in population levels to favor this reduction. In Brazil, we highlight mainly fluoridation of public water supplies and widespread use of fluoridated toothpaste as preventative measures that may be influencing the patterns of dental caries. However, we cannot exclude the important significance of broad socioeconomic factors in explaining changes in 12-year-old caries levels, as observed in industrialized countries in the 1970s and early 1980s [27].

Therefore, it is important to recognize that caries is still a problem in Brazil and in Latin America and Caribbean. It is important to consider that the figures are still alarming and indicate that a significant parcel of children and adolescents have cavitated caries lesions, even considering improvement in socioeconomic conditions, preventive measurements, access to fluorides or initiatives in education for oral health. It is also important to notice the increase in levels of inequality are usually observed in association with reduced rates of cavitated caries lesions [28]. Although these improvements are occurring, it is quite possible that in some specific groups, usually of lower socioeconomic status, there are a higher proportion of these cavities, deserving greater targeting of efforts to control the disease.

In the 2000s, caries still affects many children and adolescents in its more severe form (cavities). Moreover, the decline of caries prevalence, which was observed in the end of the 20^th^ century [5], is still occurring only for permanent teeth in the 2000s, especially among 12-year-old Brazilian children.

## Supporting Information

S1 Table “Meta-analysis of observational studies in epidemiology” (MOOSE) checklist. (PDF)

S2 Table Characteristics of each included study for primary dentition. (DOCX)

S3 Table Characteristics of each included study for permanent dentition. (DOCX)
