# Supplementary material for: Does the Decline in Caries Prevalence of Latin American and Caribbean Children Continue in the New Century? Evidence from Systematic Review with Meta-Analysis
Source: PLoS One. 2016 Oct 21;11(10):e0164903. doi: 10.1371/journal.pone.0164903 (PMC5074528; doi:10.1371/journal.pone.0164903)
Supplement: S1 Table — (PDF) [file pone.0164903.s001.pdf]

MOOSE Checklist

From: Donna F. Stroup[1], PhD, MSc; Jesse A. Berlin, ScD; Sally C. Morton, PhD; Ingram Olkin, PhD; G. David Williamson, PhD; Drummond Rennie, MD; David Moher, MSc; Betsy J. Becker, PhD; Theresa Ann Sipe, PhD; Stephen B. Thacker, MD, MSc; for the Meta-analysis Of Observational Studies in Epidemiology (MOOSE) Group. **Meta-analysis of Observational Studies in Epidemiology. A Proposal for Reporting** JAMA. 2000;283(15):2008-2012. doi: 10.1001/jama.283.15.2008

	Reported on page	Comments
**Reporting of background should include**		
Problem definition	3	
Hypothesis statement	3	
Description of study outcomes	3/4	
Type of exposure or intervention used	n/a	Outcome:prevalence (exposure not necessary)
Type of study designs used	3	
Study population	3/4	
**Reporting of search strategy should include**		
Qualifications of searchers (eg librarians and investigators)	6	
Search strategy, including time period used in the synthesis and key words	4/5 Fig. 1	
Effort to include all available studies	4/5	
Databases and registries searched	4/5	
Search software used, name and version, including special features used (eg explosion)	4/5	
Use of hand searching (eg reference lists of obtained articles)	5	
List of citations located and those excluded, including justification	Fig.2	Quantitative report
Method of addressing articles published in languages other than English	6	
Method of handling abstracts and unpublished studies	5/6	
Description of any contact with authors	n/a	
**Reporting of methods should include**		
Description of relevance or appropriateness of studies assembled for assessing the hypothesis to be tested	5/6	
Rationale for the selection and coding of data (eg	5/6	

sound clinical principles or convenience)		
Documentation of how data were classified and coded (eg multiple raters, blinding and interrater reliability)	n/a	
Assessment of confounding (eg comparability of cases and controls in studies where appropriate)	n/a	Only prevalence investigated
Assessment of study quality, including blinding of quality assessors, stratification or regression on possible predictors of study results	n/a	Only prevalence investigated
Assessment of heterogeneity	7	
Description of statistical methods (eg complete description of fixed or random effects models, justification of whether the chosen models account for predictors of study results, dose-response models, or cumulative meta-analysis) in sufficient detail to be replicated	6/7	
Provision of appropriate tables and graphics	Fig.2	
**Reporting of results should include**		
Graphic summarizing individual study estimates and overall estimate	8<br>Fig.3/4	
Table giving descriptive information for each study included	8<br>Table S2	
Results of sensitivity testing (eg subgroup analysis)	9/10	
Indication of statistical uncertainty of findings	9<br>Fig. 3/4	
**Reporting of discussion should include**		
Quantitative assessment of bias (eg publication bias)	11/14	
Justification for exclusion (eg exclusion of non-English language citations)	13	
Assessment of quality of included studies	n/a	
**Reporting of conclusions should include**		
Consideration of alternative explanations for observed results	10-15	
Generalization of the conclusions (eg appropriate for the data presented and within the domain of the literature review)	11-12/15	
Guidelines for future research	14/15	
Disclosure of funding source	16	

Transcribed from the original paper within the Support Unit for Research Evidence (SURE), Cardiff University, United Kingdom. February 2011. n/a: not applicable
