# Supplementary material for: Does the Decline in Caries Prevalence of Latin American and Caribbean Children Continue in the New Century? Evidence from Systematic Review with Meta-Analysis
Source: PLoS One. 2016 Oct 21;11(10):e0164903. doi: 10.1371/journal.pone.0164903 (PMC5074528; doi:10.1371/journal.pone.0164903)
Supplement: S2 Table — (DOCX) [file pone.0164903.s002.docx]

**S2 Table.** Characteristics of each included study for primary dentition.

| Included Studies | N | Caries Prevalence (%) | Children´s age | Country | Year of survey | Caries diagnosis criteria | Calibration of examiner |
| --- | --- | --- | --- | --- | --- | --- | --- |
| Gomes PR et al. 2004 A | 96 | 45.8 | 5 | BRAZIL | 2000 | WHO | YES |
| Gomes PR et al. 2004 B | 96 | 58.3 | 6 | BRAZIL | 2000 | WHO | YES |
| Ferreira SH et a, 2007 | 295 | 49.5 | 5 | BRAZIL | 2000 | WHO | YES |
| Sanchez-Perez L et al 2009 | 95 | 60 | 6 | MEXICO | 2001 | WHO | YES |
| Traebert JL et al. 2001 | 825 | 58.3 | 6 | BRAZIL | 2001 | WHO | YES |
| Irigoyen ME et al., 2012 | 5388 | 61.1 | 6 | MEXICO | 2001 | WHO | YES |
| Martins RJ et al 2006 A | 40 | 60 | 5 | BRAZIL | 2002 | WHO | YES |
| Cypriano S et al. 2003 | 375 | 62.7 | 5 | BRAZIL | 2003 | WHO | YES |
| Rihs LB et al 2007 | 624 | 42.6 | 5 | BRAZIL | 2004 | WHO | YES |
| Martins RJ et al 2006 B | 63 | 54.7 | 5 | BRAZIL | 2004 | WHO | YES |
| Moura Lde F et al. 2006 A | 102 | 43.14 | 5 | BRAZIL | 2004 | WHO | YES |
| Moura Lde F et al. 2006 B | 47 | 57.45 | 6 | BRAZIL | 2004 | WHO | YES |
| Ruiz LA et al., 2009 | 142 | 50.7 | 5 | BRAZIL | 2004 | WHO | YES |
| Rihs LB et al., 2008 A | 214 | 53.7 | 5 | BRAZIL | 2004 | WHO | YES |
| Rihs LB et al., 2008 B | 100 | 58 | 6 | BRAZIL | 2004 | WHO | YES |
| Meirelles MP et al., 2008 | 186 | 47.8 | 5 | BRAZIL | 2004 | WHO | YES |
| Assaf AV et al., 2006 A | 171 | 41.53 | 5 | BRAZIL | 2004 | WHO | YES |
| Assaf AV et al., 2006 B | 203 | 51.7 | 6 | BRAZIL | 2004 | WHO | YES |
| Rodríguez Vilchis LE., 2006 | 218 | 73.5 | 6 | MEXICO | 2004 | WHO | YES |
| Silva JS et al., 2006 A | 41 | 53.65 | 5 | BRAZIL | 2004 | WHO | YES |
| Silva JS et al., 2006 B | 41 | 63.41 | 6 | BRAZIL | 2004 | WHO | YES |
| Bonanato K et al 2010 | 551 | 36.1 | 5 | BRAZIL | 2005 | WHO | YES |
| Mattos V et al., 2010 | 257 | 81.7 | 6 | PERU | 2005 | WHO | YES |
| Pinheiro HHC et al., 2006 | 300 | 70.67 | 5 | BRAZIL | 2005 | WHO | YES |
| Vaz PRM et al., 2007 | 80 | 62.8 | 5 | BRAZIL | 2006 | WHO | NO |
| Melo et al., 2010 | 479 | 63.8 | 5 | BRAZIL | 2006 | WHO | YES |
| Bastos RS et al 2010 | 31 | 65.58 | 5 | BRAZIL | 2008 | WHO | YES |
| Cortellazzi KL et al 2008 | 728 | 37.8 | 5 | BRAZIL | 2008 | WHO | YES |
| Carvalho FS et al., 2009 A | 104 | 61.54 | 5 | BRAZIL | 2008 | WHO | YES |
| Carvalho FS et al., 2009 B | 50 | 62 | 6 | BRAZIL | 2008 | WHO | YES |
| Gonzalez-Martinez F, et al 2009 | 65 | 74 | 5 | COLOMBIA | 2009 | ICDAS | YES |
| Ramírez-Puerta BS et al., 2015 | 486 | 41.2 | 5 | COLOMBIA | 2009 | WHO | YES |
| Llompart G et al 2010 | 804 | 67.9 | 6 | ARGENTINA | 2010 | WHO | YES |
| Borges HC et al., 2012 A | 748 | 44.7 | 5 | BRAZIL | 2010 | WHO | YES |
| Borges HC et al., 2012 B | 638 | 45.7 | 6 | BRAZIL | 2010 | WHO | YES |
| Kramer PF et al., 2015 | 216 | 36.1 | 5 | BRAZIL | 2010 | WHO | YES |
