# Supplementary material for: Does the Decline in Caries Prevalence of Latin American and Caribbean Children Continue in the New Century? Evidence from Systematic Review with Meta-Analysis
Source: PLoS One. 2016 Oct 21;11(10):e0164903. doi: 10.1371/journal.pone.0164903 (PMC5074528; doi:10.1371/journal.pone.0164903)
Supplement: S3 Table — (DOCX) [file pone.0164903.s003.docx]

**S3 Table.** Characteristics of each included study for permanent dentition.

| Included Studies | N | Caries Prevalence (%) | Children´s age | Country | Year of survey | Caries diagnosis criteria | Calibration of examiner |
| --- | --- | --- | --- | --- | --- | --- | --- |
| Gomes PR et al 2004 | 125 | 53.6 | 12 | BRAZIL | 2000 | WHO | YES |
| Jamelli SR et al 2010 | 689 | 71.8 | 12 | BRAZIL | 2001 | WHO | NO |
| Cypriano S et al. 2008 | 2378 | 74.3 | 12 | BRAZIL | 2001 | WHO | YES |
| Tagliaferro EP et al 2008 B | 211 | 54.5 | 12 | BRAZIL | 2001 | WHO | YES |
| Bardal PA et al 2005 | 72 | 77.78 | 12 | BRAZIL | 2001 | WHO | YES |
| Cangussu MC et al 2002 | 1750 | 51 | 12 | BRAZIL | 2001 | WHO | YES |
| Sampaio FC, et al 2010 | 5782 | 67.1 | 12 | BRAZIL | 2002 | WHO | YES |
| Bueno RE, et al 2010 | 7119 | 69.3 | 12 | BRAZIL | 2002 | WHO | NO |
| Gushi LL et al 2008 B | 5782 | 67.1 | 12 | BRAZIL | 2002 | WHO | YES |
| Nomura LH et al 2004 | 169 | 57.4 | 12 13 | BRAZIL | 2002 | WHO | YES |
| Bastos JL et al. 2004 | 169 | 57.4 | 12 13 | BRAZIL | 2002 | WHO | YES |
| Freire Mdo C et al 2010 | 1947 | 64 | 12 | BRAZIL | 2003 | WHO | YES |
| Mestriner SF et al 2006 | 256 | 54 | 12 | BRAZIL | 2003 | WHO | YES |
| Antunes JL et al. 2006 | 34550 | 69 | 12 | BRAZIL | 2003 | WHO | YES |
| Traebert J et al 2005 | 444 | 55.8 | 12 | BRAZIL | 2003 | WHO | YES |
| Cypriano S et al., 2011 | 266 | 53 | 12 | BRAZIL | 2003 | WHO | YES |
| Ely HC et al., 2014a | 1177 | 50.9 | 12 | BRAZIL | 2003 | WHO | YES |
| Rihs LB et al 2010 | 309 | 61.2 | 12 | BRAZIL | 2004 | WHO | YES |
| Tagliaferro EP et al 2006 | 206 | 46.8 | 12 | BRAZIL | 2004 | WHO | YES |
| Meneghim Mde C et al 2006 | 236 | 50 | 11 12 | BRAZIL | 2004 | WHO | YES |
| Ruiz LA et al., 2009 | 173 | 58.4 | 12 | BRAZIL | 2004 | WHO | YES |
| Rihs LB et al., 2008 A | 97 | 82.5 | 11 | BRAZIL | 2004 | WHO | YES |
| Rihs LB et al., 2008 B | 149 | 73.2 | 12 | BRAZIL | 2004 | WHO | YES |
| Meirelles MPMR et al., 2008 | 195 | 57.7 | 12 | BRAZIL | 2004 | WHO | YES |
| Rihs LB et al., 2008 C | 413 | 22.8 | 12 | BRAZIL | 2004 | WHO | YES |
| Assaf AV et al., 2006 | 236 | 50 | 12 | BRAZIL | 2004 | WHO | YES |
| Rodríguez Vilchis LE et al., 2006 | 68 | 66.18 | 11 | MEXICO | 2004 | WHO | YES |
| Peres MA et al 2010 | 339 | 51.8 | 12 | BRAZIL | 2005 | WHO | YES |
| Peres MA et al 2009 | 339 | 75 | 12 | BRAZIL | 2005 | WHO | YES |
| Pereira SM et al. 2007 | 939 | 48 | 12 | BRAZIL | 2005 | WHO | YES |
| Souza ML et al 2006 | 190 | 40.9 | 12 13 | BRAZIL | 2005 | WHO | YES |
| Mattos Vela MA., 2010 | 139 | 92.8 | 12 | PERU | 2005 | WHO | YES |
| Pinheiro HHC et al., 2006 | 325 | 63.69 | 12 | BRAZIL | 2005 | WHO | YES |
| Traebert J et al 2011 | 253 | 89.7 | 12 | BRAZIL | 2006 | WHO | YES |
| Peres SH et al 2008 | 178 | 70 | 12 | BRAZIL | 2006 | WHO | YES |
| Tagliaferro EP et al 2008 A | 334 | 36.2 | 12 | BRAZIL | 2006 | WHO | YES |
| Naidu R et al 2006 | 488 | 34 | 12 | TRINIDAD E TOBAGO | 2006 | WHO | YES |
| Moura C et al., 2008 | 553 | 70.5 | 12 | BRAZIL | 2006 | WHO | YES |
| Pontigo-Loyola AP et al, 2007 | 688 | 42.6 | 12 | MEXICO | 2007 | WHO | YES |
| Benazzi AS et al., 2012 | 724 | 34.39 | 12 | BRAZIL | 2007 | WHO | YES |
| Bucker WCV et al., 2011 | 670 | 70 | 12 | BRAZIL | 2007 | WHO | YES |
| Piovesan C et al.,2011 | 792 | 39.27 | 12 | BRAZIL | 2008 | WHO | YES |
| Bastos RS, et al 2010 | 45 | 85.19 | 12 | BRAZIL | 2008 | WHO | YES |
| Piovesan C et al., 2012 | 312 | 35 | 12 | BRAZIL | 2008 | WHO | YES |
| Delgado-Angulo EK et al 2009 | 90 | 83.3 | 12 | PERU | 2009 | WHO | NO |
| Constante HM et al., 2010 | 101 | 43.5 | 12 13 | BRAZIL | 2009 | WHO | YES |
|  |  |  |  |  |  |  |  |
| Sanchez-Perez L et al 2010 | 88 | 22.7 | 11 | MEXICO | 2010 | WHO | NO |
| Pardi V et al. 2010 | 1001 | 46.8 | 12 | BRAZIL | 2010 | WHO | YES |
| Tellez M et al., 2012 | 110 | 54 | 12 | COLOMBIA | 2010 | ICDAS | NO |
| Alves LS et al., 2012 | 1528 | 38.61 | 12 | BRAZIL | 2010 | WHO | YES |
| Oliveira LB et al., 2015 | 2075 | 54 | 12 | BRAZIL | 2010 | WHO | YES |
| Frazão P et al., 2016 | 186 | 62.9 | 12 | BRAZIL | 2010 | WHO | YES |
| Jimenez-Farfan MD et al 2011 | 1139 | 72.6 | 11 12 | MEXICO | 2011 | WHO | YES |
| Ely HC et al., 2014b | 1760 | 27.2 | 12 | BRAZIL | 2011 | WHO | YES |
| Paula JS et al., 2012 | 515 | 38.83 | 12 | BRAZIL | 2012 | WHO | YES |
| Molina-Frechero N et al., 2012 | 111 | 53.2 | 11 | MEXICO | 2012 | WHO | NO |
| Tuchtenhagen S et al., 2015 | 1134 | 42.28 | 12 | BRAZIL | 2012 | WHO | YES |
| Órtiz-Leon FA, 2014 | 2375 | 90.4 | 12 | PERU | 2013 | WHO | YES |
| Freitas AR et al., 2014 | 202 | 64 | 12 | BRAZIL | 2014 | WHO | YES |
|  |  |  |  |  |  |  |  |
